# Neuroimaging Modalities Used for Ischemic Stroke Diagnosis and Monitoring

**DOI:** 10.3390/medicina59111908

**Published:** 2023-10-28

**Authors:** Jasmin J. Nukovic, Valentina Opancina, Elisa Ciceri, Mario Muto, Nebojsa Zdravkovic, Ahmet Altin, Pelin Altaysoy, Rebeka Kastelic, Diana Maria Velazquez Mendivil, Jusuf A. Nukovic, Nenad V. Markovic, Miljan Opancina, Tijana Prodanovic, Merisa Nukovic, Jelena Kostic, Nikola Prodanovic

**Affiliations:** 1 Faculty of Pharmacy and Health Travnik, University of Travnik, 72270 Travnik, Bosnia and Herzegovina; 2 Department of Radiology, General Hospital Novi Pazar, 36300 Novi Pazar, Serbia; 3 Department of Radiology, Faculty of Medical Sciences, University of Kragujevac, 34000 Kragujevac, Serbia; 4 Diagnostic Imaging and Interventional Neuroradiology Unit, Department of Neurosurgery, Fondazione IRCCS Istituto Neurologico Carlo Besta, 20133 Milan, Italy; 5 Diagnostic and Interventional Neuroradiology Unit, A.O.R.N. Cardarelli, 80131 Naples, Italy; 6 Department of Biomedical Statistics and Informatics, Faculty of Medical Sciences, University of Kragujevac, 34000 Kragujevac, Serbia; 7 Faculty of Medicine, Dokuz Eylul University, Izmir 35340, Turkey; 8 Faculty of Medicine, Bahcesehir University, Istanbul 34349, Turkey; 9 Faculty of Medicine, University of Ljubljana, 1000 Ljubljana, Slovenia; 10 Faculty of Medicine, University of Sonora, Hermosillo 83067, Mexico; 11 Department of Surgery, Faculty of Medical Sciences, University of Kragujevac, 34000 Kragujevac, Serbia; 12 Military Medical Academy, Faculty of Medicine, University of Defense, 11000 Belgrade, Serbia; 13 Department of Pediatrics, Faculty of Medical Sciences, University of Kragujevac, 34000 Kragujevac, Serbia; 14 Department of Radiology, Medical Faculty, University of Belgrade, 11120 Beograd, Serbia

**Keywords:** stroke, ischemia, CT, MR, neuroimaging

## Abstract

Strokes are one of the global leading causes of physical or mental impairment and fatality, classified into hemorrhagic and ischemic strokes. Ischemic strokes happen when a thrombus blocks or plugs an artery and interrupts or reduces blood supply to the brain tissue. Deciding on the imaging modality which will be used for stroke detection depends on the expertise and availability of staff and the infrastructure of hospitals. Magnetic resonance imaging provides valuable information, and its sensitivity for smaller infarcts is greater, while computed tomography is more extensively used, since it can promptly exclude acute cerebral hemorrhages and is more favorable speed-wise. The aim of this article was to give information about the neuroimaging modalities used for the diagnosis and monitoring of ischemic strokes. We reviewed the available literature and presented the use of computed tomography, CT angiography, CT perfusion, magnetic resonance imaging, MR angiography and MR perfusion for the detection of ischemic strokes and their monitoring in different phases of stroke development.

## 1. Introduction

Strokes are one of the global leading causes of physical or mental impairment and fatality, associated with focal CNS injury of vascular origin which precipitates neurological deficit [1]. Fundamentally, strokes are classified into hemorrhagic and ischemic strokes (ISs). ISs happen when a thrombus blocks or plugs an artery and interrupts or reduces blood supply to the brain tissue. Strokes resulting from an acute decline of vascular supply to the brain comprise a notable portion of 80% of all strokes. The predisposition aspects of these stroke subtypes are both alike and discrete. Factors such as hypertension increase susceptibility to hemorrhagic strokes, but also brings about an indirect surge in ischemic strokes that result from atherosclerosis. Moreover, hyperlipidemia, atrial fibrillation, diabetes and smoking are risk factors for intracranial and extracranial vessel atherosclerosis-originated strokes and cardioembolic strokes, respectively [2]. 

Treatment strategies for acute ischemic strokes (AISs) in stroke units are chiefly focused on revascularization and avoiding further neuronal injuries. Up-to-date medical care options for AISs are available, like the administration of IV tissue plasminogen activator (IV-tPA) for IV thrombolysis, as well as endovascular treatment (EVT) [3]. EVT, a minimally invasive operation also known as mechanical thrombectomy, is efficient for reanalyzing occluded blood vessels and results in better outcomes for large vessel occlusion (LVO) patients, removing the clot [4]. On the other hand, recombinant tPA-induced thrombolysis causes a reperfusion of the brain. Nonetheless, it is only effective in the first 4.5 h from onset; it can increase the chance of bleeding and is not worthwhile for larger infarcts, but it should be noted that EVT also has disadvantages such as access-site hematoma, infection, vasospasm, arterial perforation or dissection, symptomatic intracerebral hemorrhage, etc. [5]. Furthermore, drugs that are able to cross the blood–brain barrier (BBB) such as liposomes, hydrogels and nanomedicines are in clinical trials, and their potentials for AIS therapy are still being investigated. Owing to their short half-life, so far, clinical studies suggest that low doses of liposomes are sufficient for better motor function and dwindling the infarct volume [6,7]. They assist the administration of gaseous particles and certain drugs by enveloping them, shielding them from physiological events like degradation and elongating their half-lives [7]. As for hydrogels, they support the injured brain tissue structurally and benefit local regeneration. Still, there is insufficient evidence to recommend liposomes or hydrogels for clinical use.

The clinical management of strokes greatly benefits from various imaging modalities. For example, to exclude neoplasms and intracranial hemorrhages (ICHs), to determine the infarct core, which is beneficial for further therapy, to understand the extent of recoverable ischemic penumbras and to have real-time data on the vascular lumens, many subtypes of magnetic resonance imaging (MRI) and computed tomography (CT) are utilized. Deciding on the imaging modality depends on the expertise and availability of staff and the infrastructure of hospitals. MRI provides exceptional information and great sensitivity for smaller infarcts, diffusion-weighted imaging (DWI) produces accurate information on the volume of the core of the infarction and MRI is also proven to be more favorable in differentiating conditions that resemble strokes, such as convulsive attacks, migraines, venous infarctions and neoplasms. Still, CT is more extensively used, can promptly exclude acute cerebral hemorrhages and is more favorable speed-wise [8]. CT perfusion (CTP) imaging benefits the selection of AIS patients for EVT by facilitating core infarct volume calculations, necessitating less workflow in deducting the onset time of strokes that arise in patients that have no previous symptoms before waking up [9]. Aging ISs can be important in both clinical and medico-legal circumstances, and more accurate information can be provided through MRI. Acute ISs are defined as strokes which happen for between 24 h and 7 days, where the early hyperacute phase occurs between 0 and 6 h and the late hyperacute phase between 6 and 24 h. Subacute ISs occur in the time frame between 1 and 3 weeks, while chronic ISs last more than 3 weeks [10].

The aim of this article was to give information on the neuroimaging modalities used for the diagnosis of ischemic strokes and their monitoring in daily practice.

## 2. Methodology

### 2.1. Study Design

Our study is designed as a literature review. 

### 2.2. Search Strategy

We conducted a review of the current literature, including original articles and reviews that studied various approaches of ischemic stroke diagnostic imaging. We performed extensive searches on the Google Scholar, PubMed and ScienceDirect databases to identify relevant manuscripts. As keywords, we used “stroke”, “ischemic stroke”, and “brain attack”, “cerebrovascular accident”, combined with “imaging”, “neuroimaging”, “diagnostic”, “diagnostic modalities”, “radiological modality” and “diagnosis”.

### 2.3. Selection Criteria

Studies reporting prospective or retrospective clinical and radiological data, as well as meta-analyses involving radiological diagnostics of ischemic strokes were included. Studies not published in the English language or did not present adequate data on the neuroimaging characteristics of ischemic strokes were not included in the study. Expert opinions, book chapters and scientific meeting abstracts were also excluded.

### 2.4. Data Extraction

The search strategy and data extraction were performed independently by ten different authors who screened the retrieved papers for eligibility and analyzed the full-text articles that met the eligibility criteria. Data were extracted and analyzed using Microsoft Excel 2015.

## 3. Results

**Figure 1:**
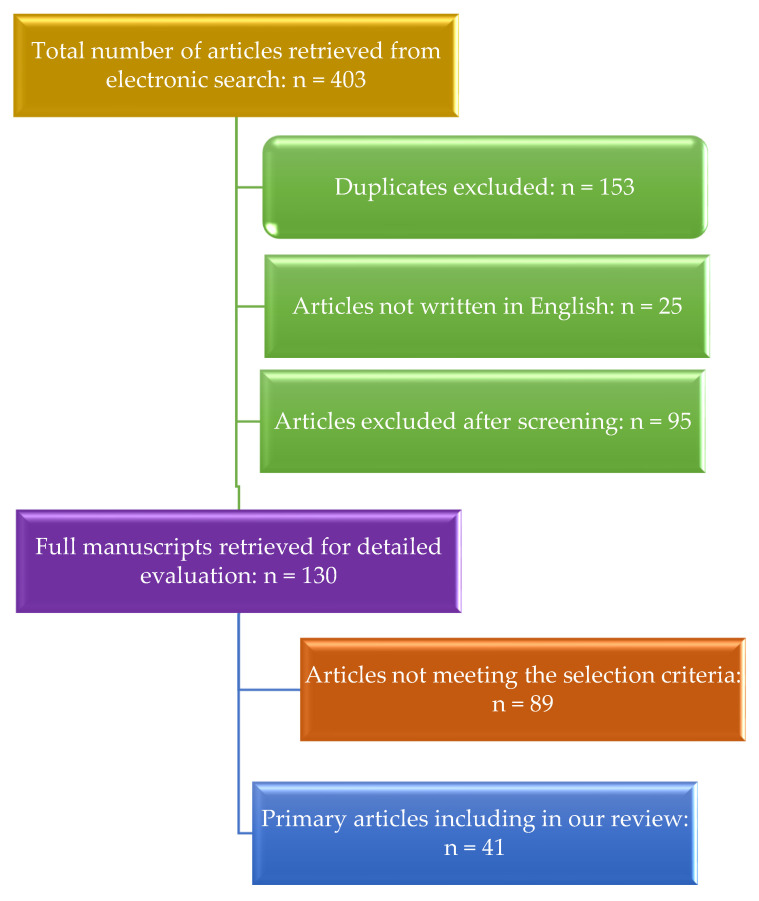
Study selection process.

The search of the literature yielded 403 articles in total. After a thorough review and assessment of the articles, we identified and included a subset of 41 papers that provided valuable insights into the neuroimaging modalities used for ischemic stroke diagnosis and monitoring, forming the basis of our review. In Figure 1, we present the study selection process.

Over time, technical development has brought the use of different modalities in the neuroimaging area. In this paper, we will present the neuroimaging modalities used for ischemic stroke diagnosis and monitoring, summarized in Table 1, with the main advantages and disadvantages of their use.

### 3.1. Non-Contrast CT

Computed tomography scans play a crucial role in diagnosing and evaluating ischemic strokes. Although other imaging modalities like DWI can offer greater sensitivity and additional information that is beneficial in later stages, the high availability, cost-effectiveness and rapid image acquisition of CT scans make them particularly suitable for the early phases of the disease [10,11]. This is especially important given that treatment options like thrombolytic therapy are most effective within the first few hours of stroke onset [12]. It should be noted that differentiation between ischemic and hemorrhagic strokes through CT is not troublesome due to the fact that hemorrhagic strokes present as hyperdense collections of blood [1].

This section will focus on the different phases of ischemic strokes and their appearances on CT scans without contrast.

#### 3.1.1. Acute Ischemic Stroke

Non-contrast computed tomography (NCCT) is a valuable tool for evaluating the initial stages of ischemic strokes. The presence of cytotoxic edema, thrombosis and cellular hypoperfusion during an ischemic event leads to observable signs on a CT scan [13,14,15,16,17,18]. These early signs are crucial for excluding intracranial hemorrhage, which is an absolute contraindication for thrombolytic therapy [14]. The most commonly seen signs include focal hypodensity, hyperdense arteries, cortical effacement, the insular ribbon sign and the obscuration of the basal ganglia [13].

The insular ribbon sign becomes evident when the insular cortex displays a reduced gray–white interface. This sign is frequently observed in middle cerebral artery (MCA) occlusions due to the insular region’s limited collateral circulation from the anterior and posterior circulations [16]. It is an extremely common sign in early ischemia, presenting itself in nearly all cases [19].

The hyperdense artery sign differs from other signs in that it reveals the thrombus obstructing the artery, rather than changes in infarcted tissue [16]. While not as easily noticeable as other early signs, it is highly specific [19].

The obscuration of the basal ganglia is frequently observed in cases of acute ischemic strokes. Since the arteries supplying the basal ganglia are primarily end-arteries, they are particularly susceptible to ischemia [13]. During acute ischemic strokes, partial disappearance or reduced differentiation may be observed on NCCT [16]. In cases where the occlusion is present in the more distal parts of the arteries, the basal ganglia might remain unaffected. Some studies indicate that only 16% of patients exhibit this sign [19].

Cortical (hemispherical) sulcal effacement is a relatively common sign, occurring in 33% of cases [20]. It indicates a partially superficial infarct, characterized by reduced contrast in the cortical sulci [18]. This phenomenon is caused by edema in the ischemic cortex [13]. When observed as an isolated sign, it indicates a better prognosis for intravenous thrombolytic therapy [19,20,21].

Focal hypodensity is a result of increased water content due to cytotoxic edema. It may present as reduced gray–white matter differentiation. While this sign is present in up to 60% of ischemic stroke cases [9], its identification can be challenging, with sensitivities for recognizing MCA territory hypodensity ranging between 60% and 85% [19].

#### 3.1.2. Aspects Score

The Alberta Stroke Program Early CT Score (ASPECTS) is a quantitative approach for identifying early stroke signs. It was developed to evaluate highly acute cases (within 3 h of symptom onset) and estimate the success rate of thrombolytic therapy [22]. Although initially designed for the anterior circulation, other models have been developed, such as pc-ASPECTS, which is used for the posterior circulation [23,24,25].

Compared to previous approaches, such as the 1/3 of MCA territory rule, ASPECTS is favored by physicians due to its higher diagnostic agreement across specialties [23,24,25].

The ASPECTS value ranges from 0 to 10. A total of 10 points indicate a normal CT scan, while 0 points suggest diffuse ischemic changes throughout the MCA territory. To calculate the ASPECTS value, starting from 10 points, one point is subtracted for each marked area affected by ischemic changes, such as hypoattenuation and swelling. These areas encompass the MCA territories M1, M2, M3, the insula, lentiform nucleus, caudate nucleus, internal capsule from ganglionic axial slices, and M4, M5 and M6 from the supraganglionic axial slices [23,24].

It is important to note that older studies examined only two cuts—one at the level of the basal ganglia and the other from the supraganglionic area [24]. Since then, common practice has changed, and typically, all axial slices are examined [22].

The relationship between treatment outcomes and the ASPECTS value has been a subject of long-standing debate. Earlier studies suggested a dichotomy between patients with a score higher than seven, who responded to treatment, and those with a lower score, who did not [25]. Recent studies indicate a linear relationship between a patient’s ASPECTS score and their response to treatment. However, a distinct cutoff point for when thrombolytic therapy becomes unviable has not yet been established, so caution should be exercised when making treatment decisions based solely on the ASPECT score [22]. Nevertheless, the ASPECTS score remains a strong predictor of functional outcomes, providing information about potential hemorrhagic transformation, recovery and response to treatment [26].

#### 3.1.3. Subacute Phase

A challenging aspect of NCCT imaging in diagnosing subacute ischemic strokes is a phenomenon known as “CT Fogging.” This fogging effect can be described as the attenuation of affected tissue returning to a “normal” state (similar to healthy brain matter) during the second and third weeks after symptom onset [27].

CT fogging is typically attributed to multiple factors, one of which is the reduction of edema and mass effect during the recovery phase [28]. Another contributing factor may be the extravasation of liquids, leading to increased blood flow and subsequently causing the fogging effect. This concept is supported by the fact that newly formed and unstable blood vessels lack a functional BBB, potentially causing contrast medium leakage if applied [29]. Studies suggest that applying a contrast medium reveals infarcted tissue during the second and third weeks, which is the usual time window for CT fogging [27].

Since CT fogging is a common occurrence (approximately 54% of cases), accurately diagnosing subacute ischemic strokes using only NCCT may be challenging. Infarcted tissue may become isodense; lesions may be underestimated, or even overlooked entirely. Consequently, ruling out a stroke cannot be easily achieved, especially if the patient presents with symptoms; in such cases, CT with contrast should be considered [28,29,30].

#### 3.1.4. Chronic Phase

As necrotic brain tissue is reabsorbed, infarcted brain tissue is replaced by cerebrospinal fluid (CSF), resulting in hypodensity. Adjacent subarachnoid and ventricular spaces may enlarge due to the negative pressure resulting from tissue loss. The infarcted area becomes clearly visible at this stage [31].

During this stage, NCCT is typically employed to monitor the recovery period and measure therapeutic success. Given that CT imaging primarily reflects structural changes in brain tissue, metrics such as infarct size are observed and correlated with clinical outcomes [32].

### 3.2. CT Angiography

In the context of an ischemic stroke, CT angiography (CTA) is used to localize the site of arterial occlusion [33] and the clot dimensions in order to plan the reperfusion treatment in an efficient way and re-establish brain circulation as early as possible [34]. Since it is accurate for the detection or large thrombi in proximal cerebral arteries [35], this particular type of occlusion can be resistant to the enzymatic breakdown from IV thrombolytic therapy [35,36]. It is important to mention that the contrast required for this study requires a wait of at least five minutes after the simple CT is performed [37].

For the study and interpretation of CT angiography, it is important to understand the division of vascular territories. This classification contains many divisions, starting with anterior and posterior circulation; the anterior cerebral artery (ACA), middle cerebral artery (MCA) and internal carotid artery (ICA) are part of the anterior circulation, whilst the posterior cerebral artery (PCA), cerebellar arteries, vertebral artery (VA) and basilar artery (BA) constitute the posterior circulation [38,39]. The ICA originates the anterior circulation vessels and is divided into four portions: cervical, petrous, cavernous and supraclinoid. On the other side, it has very particular occlusive patterns: stump, spearhead and streak/elongation; from these patterns, stumps are the most common in the cervical portion of the ICA and can be accompanied by calcifications. Streaks are the most frequent in terminus occlusion and spearheads are more common in the occlusion of the cavernous portion [40].

The ACA territory involves the medial frontal gyrus, superior frontal gyrus, the anterior part of the cingulate, the middle part of the cingulate and the corpus callosum; it also extends to the precuneus and splenium posteriorly [38]. For practical purposes, the ACA is divided in three main segments [41]:

A1: Horizontal/pre-communicating artery, which goes along the ipsilateral optic nerve and chiasm [41].

A2: Vertical/post-communicating artery. It goes into the interhemispheric fissure specifically anterior to the lamina terminalis [41].

A3: Pericallosal. It terminates in the choroid plexus [41].

The borders of the territory of the MCA extend from the superior frontal sulcus to the middle occipital gyrus; some of the structures involved in the territory are the anterior portion of the middle frontal gyrus, the angular gyrus and posterior involvement from the parietal lobe, inferior occipital gyrus, putamen, globus pallidus and the insula [38]. For the radiological study of the MCA, the artery is divided in four segments [42]:

M1: Sphenoidal or horizontal segment; it has its origin in the bifurcation of the carotid artery and extends laterally in the axial plane to the Sylvian fissure. It eventually divides into two trunks that, when they reach the limen insulae, turn 90 degrees. This turn is known as the genu.

M2: Insular segment. It begins its trajectory at the genu from the previous segment, composed of the trunks and its branches that circulate in the insulae, directed to the insular circular sulcus in its most distant part to make another turn to surround the opercula.

M3: Opercular segment. It originates from the circular sulcus’s most distal part; the branches go along the temporal and frontoparietal opercula until the lateral edge of the Sylvian fissure.

M4: Cortical segments. They begin in the lateral edge of the Sylvian fissure, where the branches make another turn to an inferior or superior direction towards the cortical surface, where they terminate [42]. 

The VA is divided into extracranial and intracranial segments; its branches supply the medulla and spinal cord anterior surfaces. Besides originating the PICA and the BA at the conjunction of the intracranial portion, the BA perforating branches irrigate the brain stem; besides that, the BA originates the SCA, AICA and PCA [43].

The cerebellum is irrigated by the SCA, AICA and PICA; specifically, the SCA and AICA originate from the BA, the AICA supplies anterior–inferior cerebellum, whilst the SCA vascularizes the superior vermis and superior cerebellum. On the other hand, the PICA irrigates the lower vermis, lower medulla and posterior–inferior cerebellum [43].

The PCA constitutes the terminal branch of the BA; in its territory, the lateral border is delimited to the lateral part of the frontal gyrus and extends to the inferior occipital gyrus, middle occipital gyrus and superior occipital sulcus. This territory includes interhemispheric surfaces of the occipital lobe such as the lingual gyrus, calcarine gyrus and lower half of the cuneus; it also includes the thalamus and midbrain [38]. The PCA also has four segments [39]:

P1: Pre-communicating segment. It extends from the end of the basilar artery (BA) and ends in the posterior communicating artery (PCOM).

P2: Post-communicating segment. It has its origin at the PCOM and ends when it enters the quadrigeminal cistern.

P3: Quadrigeminal. This follows the course of the quadrigeminal cistern.

P4: Cortical. This goes along the calcarine fissure and becomes the calcarine artery [39].

Another aspect that needs to be evaluated in CT angiography is the percentage of blockage in the artery, where stenosis is defined as a narrowing of the vessel, while occlusion refers to a total blockage; the severity of the stenosis will be classified according to the percentage, where <50% is mild stenosis, >50–<70 is moderate stenosis, 70–89% is severe stenosis and 90–99% is very severe stenosis [44].

For reperfusion therapy, CT angiography has clinical relevance in selecting the type of treatment that patients will receive for ischemic strokes, since a key point in the selection is the size and location of the occlusion site [35,36]. Large and proximal arterial occlusions (ICA, M1, M2 or BA) can be associated with negative outcomes and a need for extended care, besides having lower recanalization rates with IV thrombolysis; therefore, a mechanical thrombectomy would represent a better option for these patients [45].

### 3.3. CT Perfusion

Besides being a diagnostic tool, CT perfusion plays an important role when it comes to selecting patients that could be candidates for reperfusion therapy [46], since it is capable of determining the following variants: mean time transit (MTT), time to peak (TTP), cerebral blood flow (CBF) and cerebral blood volume (CBV). It provides an accurate delimitation of the infarct core and penumbra area; this way, the areas that can be saved can be visualized [47]. However, it is important to take into account the limitations of this technique, such as the overestimation of the infarct core and a 50% false negative rate for lacunar strokes [48].

The information that CT perfusion seeks to capture is the dynamic of a contrast bolus passing through brain tissue, which includes its increase, peak and decrease in the area of interest, in order to obtain a quantification of the tissue with hypoperfusion. The following parameters are representative of different aspects of the blood flow that goes into the brain [47]:

CBF: Describes the blood flow volume of blood streaming into a unit of brain mass throughout a unit of time that is measured in mL/100 g/min.

CBV: Refers to the vascularized fraction of a tissue, reported in millimeters/100 g.

MTT: Represents the time it takes, on average, for the contrast bolus to cross the capillary beds; the MTT unit is in absolute seconds. This variant in particular is dependent on the two previous variants (CBF and CBV) since it is calculated by the formula MTT = CBV/CBF.

TTP: Reports the average time that the contrast medium takes to reach its highest level in the area of interest [47].

To this day, there is no standardized classification between the different processors to define the core and penumbra, since vendors establish their variations to define infarct cores and penumbras in their software [49]. However, it is the match or mismatch between the CBV and CBF with the MTT and TTP that will determine if the area is a penumbra or infarct core; this means that, if all the variants match on having altered levels, as indicated in Table 2, it is classified as an infarct. On the other hand, if there is a mismatch because the CBV or CBF only have mild alterations but the MTT and TTP are increased, the phenomenon is classified as a penumbra, since it is considered that there is salvageable tissue left [8].

**Figure 2:**
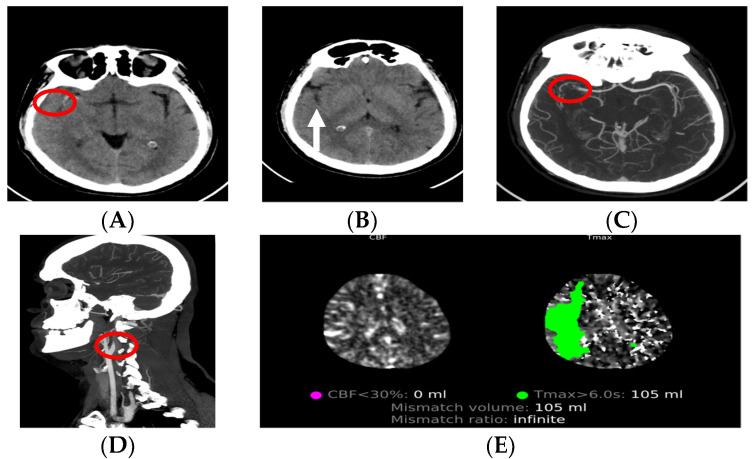
Patient, male, 48 years old, presented with left hemiplegia and dysarthria at the emergency department, after which a neurologist ordered CT diagnostics. (**A**,**B**) Non-contrast CT which shows MCA hyperdensity (red circle), loss of right insular ribbon (white arrow) and ASPECT 9. (**C**,**D**) CT angiography, axial and sagittal, showing tandem occlusion of origin of R-ICA (dissection) and R-MCA (both in red circles). (**E**) CT perfusion showing mismatch volume of 105 mL.

CT perfusion allows patients who are outside the time window for reperfusion therapy (>4.5 h for intravenous thrombolysis and >6 h for mechanical thrombectomy (MT)) to have the opportunity to receive treatment, if there is a penumbra area left that is viable [47,50]. Under the criteria for CT perfusion, an MT can be performed in patients with an infarct core of ≤70 mL and large vessel occlusion [36]. The use of CT, CT angiography and CT perfusion in daily practice (experience from the Stroke Center, AORN Cardarelli, Naples, Italy) is presented in Figure 2.

### 3.4. MRI in Ischemic Stroke

Magnetic resonance imaging is a non-invasive imaging modality that can be used to study the body’s soft tissues [51,52]. To visualize the internal organization of an anatomical region, MRI takes advantage of the magnetic properties of the hydrogen nuclei from water molecules [52]. Although MRI is more specific and sensitive than CT, it is not commonly used for the initial diagnosis of an acute ischemic stroke. MRI is a less frequently used option as a primary imaging modality, mostly due to its greater price, extended scan time, lack of availability and more difficult workflow. The use of MRI in the initial assessment of acute ischemic strokes is limited and reserved only for unique indications. However, after the emergency setting, brain MRI can give more precise diagnostic information to improve further care [53]. 

Specific parts of the brain are visualized using different MRI sequences [52]. T1- and T2-weighted imaging (T1WI and T2WI) are the two MRI sequences that are most frequently used [54]. These two conventional MRI sequences, together with non-contrast CT, represent a standard imaging protocol in the imaging of a stroke and have been used mainly to exclude hemorrhages [55]. In T1WI, fluid or water-containing tissues appear dark, while fatty tissues appear bright. In T2WI, fatty tissues appear dark, and fluid or water-containing tissues appear bright [52]. The gradient-echo T2* (GRE T2*wi) sequence is a common additional MRI sequence that gives a correct assessment of hemorrhagic alteration [56,57]. Susceptibility-weighted imaging (SWI) sequences are akin to the GRE T2*wi sequence, but has better sensitivity to detect hemorrhages. Fluid attenuation inversion recovery (FLAIR) is another MRI sequence that is akin to T2WI, with the only exception being that the cerebrospinal fluid appears darker while abnormalities stay bright [52]. FLAIR imaging most importantly helps to establish the age of infarctions, and its essential clinical use is to recognize acute ischemic infarcts within therapeutic intervention time windows [58]. Diffusion-weighted MR imaging (DWI) is a sequence that recognizes the movement of water molecules [52]. It is frequently used in the diagnosis of acute brain infarctions because of its ability to reveal cytotoxic edema [59]. Ischemic brain tissue appears as a bright area or a spot on the image as a result of restricted water movement in the cells [54]. The apparent diffusion coefficient (ADC) quantitatively expresses the degrees of diffusion, and its lowered values indicate a restriction in diffusion [55,59]. However, DWI may be, at times, incapable of identifying small infarcts and can misidentify reversible injuries as irreversible. To improve image accuracy, DWI is frequently used in conjunction with perfusion-weighted imaging (PWI). PWI measures perfusions of the cerebrum using the evaluation of hemodynamic parameters like mean transit time, cerebral blood flow and cerebral blood volume [52]. The DWI/PWI mismatch concept has been used to define the presence of hypoperfused ischemic penumbra in PWI and the infarct core in DWI [52,60,61]. It has been tested as a thrombolysis selection marker and can also provide a prediction of the final infarct area, which can be used to reduce variability between cases in studying the treatment outcome [62]. The time-of-fight (TOF) sequence is used for depicting the level of proximal vessel occlusion. It has high sensitivity and specificity to detect blood flow absences; however, there is a risk of artifacts due to the MR signal being generated by blood flow [63,64]. Another disadvantage is that it is not able to give a direct visualization of the thrombus [63]. The location of occlusions can be misidentified because of lower sensitivity to slow flow, or the stenosis degree can be overestimated when there are hemodynamic changes present [63,65]. Analyzing the intensities of the signal in different sequences (DWI, ADC and FLAIR) can help to differentiate between different stages of ischemic strokes [17]. 

#### 3.4.1. Early Hyperacute

Because of a decrease in the ADC, DWI can detect ischemic tissue alterations from a matter of minutes to a couple of hours following arterial occlusion. Hyperintense tissue or increased DWI signals and reductions in the ADC mean irreversible ischemia [17]. In other MRI sequences, the affected tissue looks normal [55]. No hyperintensity in FLAIR and increased signals in DWI suggest that the stroke happened less than 4.5 h prior to imaging [17]. At this stage, alterations in blood flow can be seen in MRA and thromboembolism can be detected using SWI [66].

#### 3.4.2. Late Hyperacute

A signal with elevated T2 intensity is usually detected after six hours. The signal is initially more prominent in FLAIR and not in the conventional T2 sequence. Over the next few days, these changes continue to aggravate. The decrease in T1 becomes evident after 16 h and carries on [55]. Twenty-four hours after occlusion, 90% of infarctions can be seen in T2WI, but only 50% are detected in T1WI. Furthermore, T1WI and T2WI have presented a high false negative rate within the initial 24 h after stroke onset. However, when combing data from T1WI, T2WI and DWI, they greatly correlate with the histology of tissue [55]. 

#### 3.4.3. Acute

The ADC values are initially reduced; later, they start to pseudonormalize, and after the first week, the ADC values start to rise [17,55]. The infarcted parenchyma continues to exhibit a high DWI signal. In FLAIR and T2, the infarction area remains hyperintense, with T2 signals gradually intensifying during the first four days. T1 signals stay low. When using T1 C+, contrast enhancement of the cortex is usually seen 5 days after the onset of the stroke. Arterial and meningeal enhancements are even less frequent and can also be visible during the acute stage [55].

#### 3.4.4. Subacute + Chronic Ischemic Strokes

As stated by International Stroke Recovery and Rehabilitation Roundtable 19, the subacute stages of ischemic strokes are indicated to be between a week and 6 months post-stroke (broken down to early and late at 3 months) and the chronic stage is affirmed to be after 6 months of onset [67]. DWI and ADC imaging modalities compared to conventional MRI and CT can present with alterations from minutes to hours. The hyperdensity seen in DWI images starts to diminish after a week, while the ADC values are lessened upon the onset of ischemia, and after 7–10 days, images begin to appear brighter, resulting in a relatively normal ADC, also referred to as the “pseudonormalization of ADC”. This sign can help to distinguish acute infarcts from subacute and chronic infarcts; the latter appear to be more hypodense in DWI and more hyperdense in ADC images with time [68]. 

DWI in the subacute phases may remain hyperintense for longer through the means of the firmly elevated T2/FLAIR signals, an effect called the “T2 shine through” [67]. Brighter DWI images result from T2 taking longer to be cleansed from tissues, not from the restriction of diffusion, which, if that were the case, would be proven through ADC hypointensity [69]. Likewise, the “Fogging phenomenon” is seen in T2 sequences of cerebral infarctions in nearly half of patients between 1 and 5 weeks, with a median of 10 days after lesion onset, marked by the cortical appearance almost returning to normal [67,70]. FLAIR sequences promote the bright appearance of ischemic areas through the suppression of cerebrospinal fluid intensity with prolonged T2. The intensity of FLAIR images reach their maximum in chronic infarcts [67,71]. Contrast enhancement in cortical regions may carry on for 2–4 months [71]. High T1-weighted image signals detected in the hyperacute phases start to transform into hypointense signals after 16 days, parenchymal enhancement is prevalent following BBB breakdown and cortical intrinsic laminar necrosis-induced hyperintensity may also be seen [67,71,72]. The spot of vessel occlusion determined through MRA, which has contrast-enhanced and non-contrast-enhanced techniques, may benefit the prediction of the final outcome associated with infarction and penumbra generation [15,73]. For monitoring of the final infarct range after 1 to 3 months, T1- and T2-weighted images are utilized [15].

**Figure 3:**
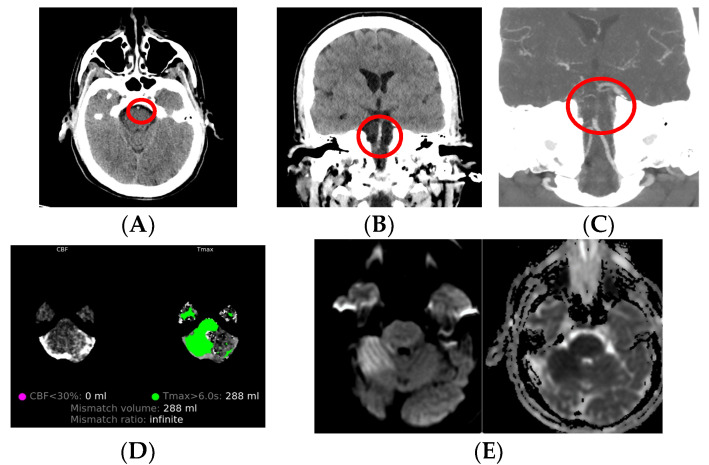
Male patient, 45 years old, presented with vertigo and disorientation at the emergency department, after which a neurologist ordered neuroimaging. (**A**,**B**) Non-contrast CT shows hyperdense basilar artery (red circle). (**C**) CT angiography shows occlusion of basilar artery. (**D**). CT perfusion shows mismatch volume of 288 mL and marks ischemia zone. (**E**) MRI in sequences DWI and ADC show restricted diffusion in the right cerebellar region, marking acute ischemia.

The use of CT, CT angiography and CT perfusion, combined with MRI in daily practice (experience from the Stroke Center, AORN Cardarelli, Naples, Italy), is presented in Figure 3.

### 3.5. MR Angiography

Even though CTA is used as the gold standard for the detection of large vessel occlusion in acute strokes, MR angiography (MRA) is more than beneficial in patients with contraindications of intravenous contrast media used in CTA. However, MRA images are flow-dependent and may be inaccurate, unlike CTA, which presents the true anatomy of the vessel lumen [74].

MRA has benefits in the determination of stenosis severity, as well as vascular occlusion and collateral flow in AIS patients. Contrast-enhanced MRA and 3D time-of-flight (TOF) are useful for distinctions between surgical and non-surgical carotid stenosis. Three-dimensional TOF MRA has high accuracy in the evaluation of proximal stenosis/occlusions (unlike distal), while the 2D phase contrast MRA technique has an important place in the evaluation of Willis circle collateral flow schemes [75].

### 3.6. Magnetic Resonance Perfusion Imaging

MR perfusion (MRP) assesses brain tissue perfusion levels. It creates perfusion maps, CBF, CBV, MTT and time to peak/time to maximum. [74]. In most MTP protocols, gadolinium is applied i.v., after which GRE T2*wi is performed, although sometimes T1-WI. However, evidence from the literature suggests that CTP has a better determination of penumbra compared to MRP. Trials have shown that MRP is beneficial for the determination of patients with less chances of undesirable outcomes after reperfusion treatment, having Tmax > 8 s as a threshold [76].

## 4. Future Directions

The future of neuroimaging lies in artificial intelligence. It is a promising field, since it increases efficiency and reduces errors, which are particularly significant in pathological entities such as acute ISs when timing is important for treatment options and, consequently, outcome. MRI consumes time and is not available in all stroke units, and artificial intelligence suggests the use of compressed sensing since it gives a scan with a singular viewpoint. The optimization between magnetic resonance imaging and compressed sensing is one of the most promising neuroimaging applications, with important implications in strokes. Also, the development of new software which provide an automatic ASPECT score and quantitative assessment of CT perfusion have enormous clinical benefits, since they reduce time consumption and help to assess proper candidates for thrombectomy [77]. Ethical considerations need to be assessed in this field in the future, as well as more clinical studies. 

## 5. Conclusions

The use of neuroimaging in ischemic stroke patients is crucial in order for patients to receive the correct diagnosis and receive optimal treatment in a timely manner. To this day, CT and MRI have been proven to have high specificity in ischemic stroke patients, alongside CT and MR angiography and CT and MR perfusion techniques. Each of the techniques provide valuable information for clinicians in terms of the stroke type and cause, its location, size, core and penumbra, collateral flow, etc., which are of great importance for choosing the best treatment option and achieving optimal outcomes. The future of neuroimaging lies in the hands of artificial intelligence, whose solutions will aid in increased efficiency and reduced errors for all medical professionals working with stroke patients.

## Figures and Tables

**Table 1:** Summary of neuroimaging modalities used for ischemic stroke diagnosis and monitoring.

	Advantages	Disadvantages
Non-contrast CT	High availability, cost-effectiveness, and rapid image acquisition; no contrast.	Ionizing radiation. Limited in posterior fossa and small lesions, as well as hyperacute and acute IS.
CT angiography	Locates source of thrombi or emboli and the clot dimensions in order to plan the reperfusion treatment.	Ionizing radiation. Contrast contraindications.
CT perfusion	Provides an accurate delimitation of the infarct core and penumbra area and thus good selection of patients for reperfusion treatment.	Ionizing radiation. Limited availability.
MRI	No radiation; greater sensitivity than CT; better detection of small lesions in comparison to CT.	Slower than CT; higher cost; limited availability.
MR angiography	Locate source of thrombi or emboli; no contrast.	Flow-dependent images may be inaccurate, unlike CTA which presents true anatomy of vessel lumen.
MR perfusion	Assesses brain tissue perfusion level.	Higher cost; limited availability; use of contrast.

**Table 2:** CT perfusion variant thresholds for penumbra and infarct core.

Variant	CBF	CBV	MTT	TTP
Penumbra	12–20 mL/100 g/min	>2 mL/100 g	145%	Increased
Infarct core	<10–12 mL/100 g/min	<2 mL/100 g	145%	Increased

## Data Availability

Data supporting the reported results are available on request from the corresponding authors.
